# Monkeypox and oral lesions associated with its occurrence: a systematic review and meta-analysis

**DOI:** 10.12688/f1000research.137363.1

**Published:** 2023-08-10

**Authors:** Hiroj Bagde, Ashwini Dhopte, Ferdous Bukhary, Naif Momenah, Fatema Akhter, Okba Mahmoud, Krishna Prasad Shetty, Maher AL Shayeb, Huda Abutayyem, Mohammad Khursheed Alam

**Affiliations:** 1 Periodontology, Rama University, Kanpur, Uttar Pradesh, 208024, India; 2 Oral Medicine and Radiology, Rama University, Kanpur, Uttar Pradesh, 208024, India; 3 Preventive Dental Sciences, Dar Al Uloom University, Riyadh, Riyadh Province, 13314, Saudi Arabia; 4 Riyadh Second Health Cluster, Saudi Ministry of Health, Riyadh, Riyadh Province, 13314, Saudi Arabia; 5 Surgical and Diagnostic Sciences, Dar Al Uloom University, Riyadh, Riyadh Province, 13314, Saudi Arabia; 6 Clinical Science Department, College of Dentistry, Ajman University, Ajman, Ajman, United Arab Emirates; 7 Orthodontic Division, Preventive Dentistry Department, Jouf University, Sakaka, Al Jowf, 72345, Saudi Arabia

**Keywords:** Monkeypox; Oral lesions; Outbreak; Systematic review; Zoonotic virus

## Abstract

**Background:** A zoonotic, double-stranded DNA virus belonging to the genus Orthopoxvirus, the monkeypox virus is most common in tropical regions of Central and West Africa. The frequency of monkeypox cases, however, has sharply climbed globally since May 2022. **Objectives:** To establish the threat of monkeypox in terms of the oral lesions caused in sufferers. **Materials and methods:** After a thorough study of the literature identified in the PubMed, Web of Science, and Cochrane library databases using the PRISMA framework, 103 papers were found. Using inclusion and exclusion criteria, we chose research that was relevant for our review before shortlisting 14 papers that conformed to the review's guidelines. **Results:** In the 14 selected studies, it was found that oral lesions were among the first clinical signs of a monkeypox affliction, with ulcers on the dorsal surface of tongue lips being the most common areas affected. **Conclusion:** The rarely observed oral lesions of monkeypox infection may help in the diagnosis and management of this condition. It is critical to keep in mind that recognising and detecting oral lesions in monkeypox patients opens the door to more research and efficient patient management.

## Introduction

The monkeypox virus is an uncommon zoonotic disease that causes monkeypox. Both the smallpox and monkeypox viruses are members of the Orthopoxvirus genus in the Poxviridae family. In the Democratic Republic of the Congo, the first case of monkeypox on a human was documented in 1970. ^1^ Since that time, monkeypox has spread over western and central Africa. Up until recently, 47 non-endemic nations in Europe, North and South America, Asia, North Africa, and Australia had reported cases of the disease. More than 4,100 confirmed cases had been reported as of 2022, the count increasing by the minute. Notably, 87% of occurrences have happened in nations in Europe. ^2^ It is thought that the termination of smallpox vaccination, which offered some cross-protection against monkeypox but was not the primary cause of the new outbreak, led to an increase in human-to-human transmission. Generally speaking, human-to-human transmission happens when contaminated objects including linens, bedding, electronics, and clothing come into direct physical contact with an infected person's ulcerated skin or mucosa, respiratory droplets, or both. Through the placenta, pregnant women can spread the virus to unborn children. ^3^ Monkeypox virus airborne transmission is still a contentious topic. Even if it does, it could not be the primary method of transmission. The incubation period for monkeypox infection lasts for 7–14 days or 5–21 days and is not contagious. Patients do not have any symptoms during this time. ^4^

Table 1 represents the clinical characteristics of monkeypox as generally observed in sufferers. Notably, lesions in patients with monkeypox in non-endemic locations are more confined and have a different distribution of rashes, whereas symptoms in patients with monkeypox in endemic regions are more severe and result in a certain proportion of fatalities. There have been no reports of patient deaths from non-endemic regions up to this point. ^5^

**Table 1.** Clinical presentation of monkeypox generally exhibited in patients.

*Variable assessed*	*Variable characteristic*
Initial site of infection	Face
Period of illness	2-4 weeks
Time of exposure of clinical signs after infection	5-21 days
Presence of lymphadenopathy	Positive
Dermatologic appearance in palms and soles	Positive
Duration of fever before appearance of rashes	1-3 days

The beginning phase of monkeypox includes a fever, chills, headache, backache, myalgia, asthenia, and lymphadenopathy. Patients that are infected may be contagious during the prodromal stage. It's interesting to note that the fundamental distinction between the symptoms of monkeypox and smallpox is that smallpox does not result in lymphadenopathy but monkeypox does. Submandibular, cervical, axillary, and inguinal lymph nodes may be bilaterally or unilaterally affected in monkeypox. ^6^ Lesions may form in the mouth, oropharynx, or throat after the prodrome before spreading to the skin. The face and extremities, such as the palms of the hands and soles of the feet, are typically where the skin rash is more intense. The lesions develop in stages, going from macule to papule to vesicle to pustule to crust. Until all lesions have crusted over, patients are deemed contagious. The sores are frequently characterised as hurting initially, then becoming irritating. After scabs have peeled off, scars with hyper/hypopigmentation may develop. Usually, the disease lasts 2-4 weeks. Patients in the latest outbreak's non-endemic areas present with unusual symptoms that are very dissimilar from those in western and central Africa. Genital, perianal, and perioral/oral rash, fever, lymphadenopathy, and swallowing pain are a few of these. Lesions on the oral mucosa and vaginal region may first emerge before or without spreading to other body areas, pointing to sexual contact as the likely mode of transmission. ^7^ Many individuals first had pustules before becoming feverish. Patients with a few small, isolated skin lesions sometimes show no signs of pain. It's interesting to note that the same people can have lesions in various stages. Pain, haemorrhage, proctitis, and tenesmus can all result from anus and rectum lesions.

Patients in the endemic area have never before reported having these symptoms. Patients in the current outbreak locations generally experience milder symptoms than those in the endemic zone. There haven't been many documented hospitalizations, and the two most common causes were pain management and subsequent infection care. ^8^ Prior to skin rashes, monkeypox might present with oral and oropharyngeal lesions. It is stated that mouth sores, along with fever and enlarged lymph nodes, are common symptoms in monkeypox patients. Notably, the CDC stated that lesions in the mouth and on the tongue were seen in 70% of people. These findings imply that the virus can be carried in the saliva and spread via oral-skin and/or oral-anogenital contact. Therefore, it's crucial for dentists and other dental professionals to be aware of and able to identify monkeypox oral lesions. Starting with macule, papule, vesicle, and pustule, the progression of oral lesions should resemble that of skin lesions. ^9^

Ulceration with pseudomembrane occurs after the roofs of the vesicle or pustule break off. A male patient who was a returning tourist from Nigeria experienced right cervical lymphadenopathy, multiple 2-4-mm pustules, and central umbilication of the skin, particularly on the face, neck, and hands. The oral lesions were described as a single intact pustule on the lower labial mucosa and a few round 2-3 mm erosions on the mucosa, indicating that the original vesicles or pustules have already broken off. ^9^

Hence, by the means of this systematic review and subsequent meta-analysis, we aimed to establish the threat of monkeypox in terms of the severity of oral lesions that are caused due to it.

## Methods

### Protocol employed

This systematic review was performed as per the Preferred Reporting Items for Systematic Review and Meta-analysis (PRISMA) strategy and rules from the Cochrane group and the book Orderly reviews in Health care: Meta examination. ^10^ We also utilised the PICO strategy to identify and evaluate the relationship between monkeypox and oral lesions. The population of interest included individuals with confirmed cases of monkeypox. The intervention of interest was the presence of monkeypox, while the comparison group consisted of individuals without monkeypox. The outcome of interest was the occurrence of oral lesions associated with monkeypox.

### Review hypotheses

This systematic review aimed to analyse, by the means of selecting studies, to review the correlation between the incidence of monkeypox and the oral lesions occurring in patients because of it at various stages of the disease.

### Study selection

**Figure 1.**
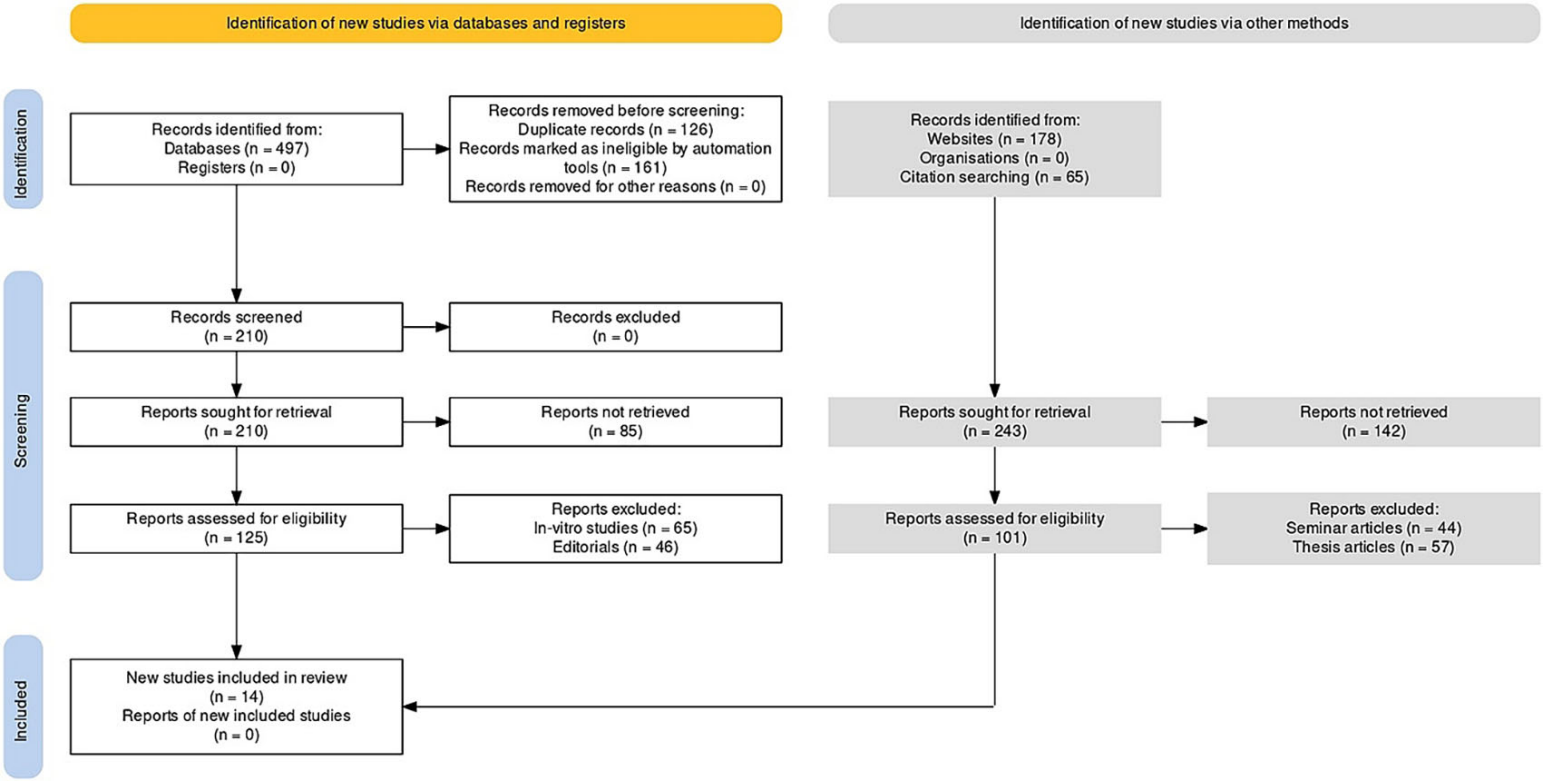
Representation of selection of articles through PRISMA framework.

There were a total of 103 documents discovered after extensive search on the online journals and 54 of the papers were selected initially. Following that, 22 similar/duplicate articles were eliminated, which resultantly made 32 separate papers available at first. The abstracts and titles of submissions were then reviewed, and a further 18 papers were eliminated. Finally, 14 documents that met the inclusion and exclusion criteria were chosen, which included study articles and randomised/non-randomised control trials ( Figure 1).

### Eligibility criteria

For this systematic review, we employed the PICOS (Participants, Interventions, Comparison, Outcome, Study design) framework for assessment of studies fit for our investigation.

The following were excluded from the scope of our systematic review: incomplete data, individuals in whom antimicrobial treatment had begun only recently, seminar presentations, scholarly articles, placebo controlled studies, and opinion articles.

Since the literature available on this topic is quite scant in volume, we did not limit our search in terms of the time period when the studies were published i.e. we took into account all the papers that were published with context to our topic (where the number of papers itself was found to be quite sparse in number).

Placebo-controlled studies were not included in the analysis. Also excluded were literature reviews and cases published in languages other than English.

### Search strategy

Using relevant keywords, reference searches, and citation searches, the databases PubMed-MEDLINE, Web of Science, Cochrane, and Scopus were all searched. “Monkeypox,” “Oral lesions,” “Zoonotic viruses” and “Oral manifestations” were the search terms used to access the database.

### Data selection and coding

Two independent reviewers located the relevant papers by using the right keywords in various databases and online search tools. The chosen articles were compared, and a third reviewer was brought in if there was a dispute.

**Figure 2.**
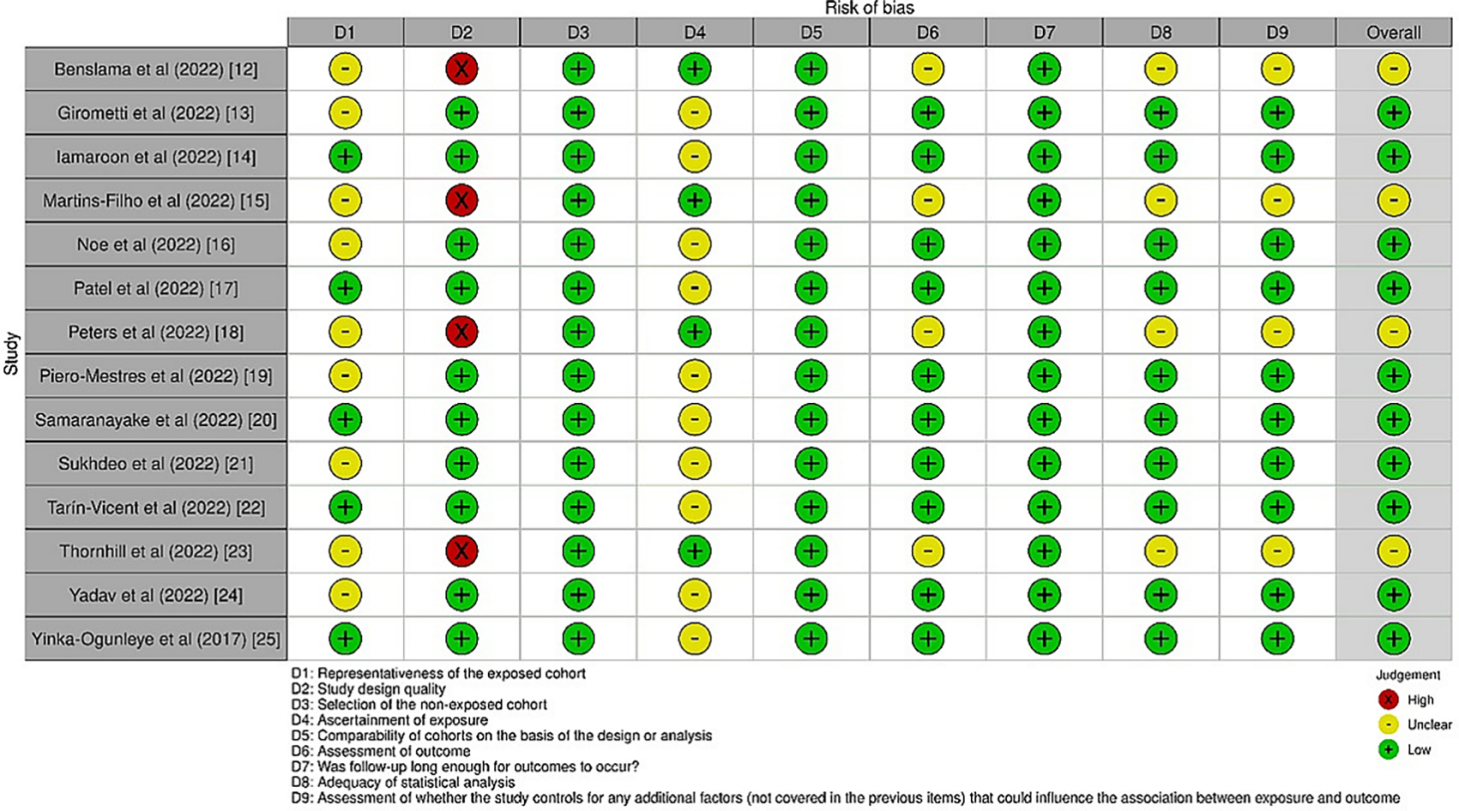
Risk analysis across selected studies.

After choosing the articles, the same two reviewers independently extracted the following data: author, year of publication, country, kind of publication, study topic, population demographics (n, age), outcome measure(s), relevant result(s), and conclusion (s). The data was compared and any differences were discussed with the third reviewer. The evaluation of risk analysis was also performed, the detail of which have been furnished in Figure 2.

### Risk of bias assessment

The AMSTAR-2 protocol ^11^ was employed for the assessment of the risk of bias in our selected studies. As a critical evaluation tool for systematic reviews, AMSTAR 2 joins a number of other instruments that have been published for this purpose. It consists of a 16-point checklist, as shown in Table 2 below. The development of the original AMSTAR tool was based on two instruments that have received a lot of attention. Two instruments that have been released are exact copies of the original AMSTAR. The domains listed in the Cochrane risk of bias instruments for systematic reviews are identified by the AMSTAR 2 risk of bias items. These represent an agreement that was reached after input from more than 30 methodology specialists in each case.

**Table 2.** AMSTAR-2 16-point checklist of risk of bias assessment in selected studies.

*Studies selected*	*Question and inclusion*	*Protocol*	*Study design*	*Comprehensive search*	*Study selection*	*Data extraction*	*Excluded studies justification*	*Included study details*	*Risk of bias*	*Funding sources*	*Statistical methods*	*Risk of bias in meta-analysis*	*Risk of bias in individual studies*	*Explanation of heterogeneity*	*Publication bias*	*Conflict of interest*
Benslama et al (2022) ^12^	Yes	Yes	Yes	Yes	Yes	No	No	No	Yes	N/A	Yes	Yes	Yes	Yes	Yes	Yes
Girometti et al (2022) ^13^	Yes	Yes	Yes	Yes	Yes	No	No	No	Yes	N/A	Yes	Yes	Yes	Yes	Yes	Yes
Iamaroon et al (2022) ^14^	Yes	Yes	Yes	Yes	Yes	No	No	No	Yes	N/A	Yes	N/A	Yes	Yes	Yes	Yes
Martins-Filho et al (2022) ^15^	Yes	Yes	Yes	Yes	Yes	No	No	No	Yes	N/A	Yes	Yes	Yes	Yes	Yes	Yes
Noe et al (2022) ^16^	Yes	Yes	Yes	Yes	Yes	No	No	No	Yes	Yes	Yes	Yes	Yes	Yes	Yes	Yes
Patel et al (2022) ^17^	Yes	Yes	Yes	Yes	Yes	No	No	No	Yes	Yes	Yes	Yes	Yes	Yes	Yes	Yes
Peters et al (2022) ^18^	Yes	Yes	Yes	Yes	Yes	No	No	No	Yes	N/A	Yes	Yes	Yes	Yes	Yes	Yes
Piero-Mestres et al (2022) ^19^	Yes	Yes	Yes	Yes	Yes	No	No	No	Yes	N/A	Yes	Yes	Yes	Yes	Yes	Yes
Samaranayake et al (2022) ^20^	Yes	Yes	Yes	Yes	Yes	No	No	No	Yes	N/A	Yes	N/A	Yes	Yes	Yes	Yes
Sukhdeo et al (2022) ^21^	Yes	Yes	Yes	Yes	Yes	No	No	No	Yes	N/A	Yes		Yes	Yes	Yes	Yes
Tarín-Vicent et al (2022) ^22^	Yes	Yes	Yes	Yes	Yes	No	No	No	Yes	N/A	Yes		Yes	Yes	Yes	Yes
Thornhill et al (2022) ^23^	Yes	Yes	Yes	Yes	Yes	No	No	No	Yes	N/A	Yes	Yes	Yes	Yes	Yes	Yes
Yadav et al (2022) ^24^	Yes	Yes	Yes	Yes	Yes	No	No	No	Yes	N/A	Yes	Yes	Yes	Yes	Yes	Yes
Yinka-Ogunleye et al (2017) ^25^	Yes	Yes	Yes	Yes	Yes	No	No	No	Yes		Yes	Yes	Yes	Yes	Yes	Yes

## Results

**Figure 3.**
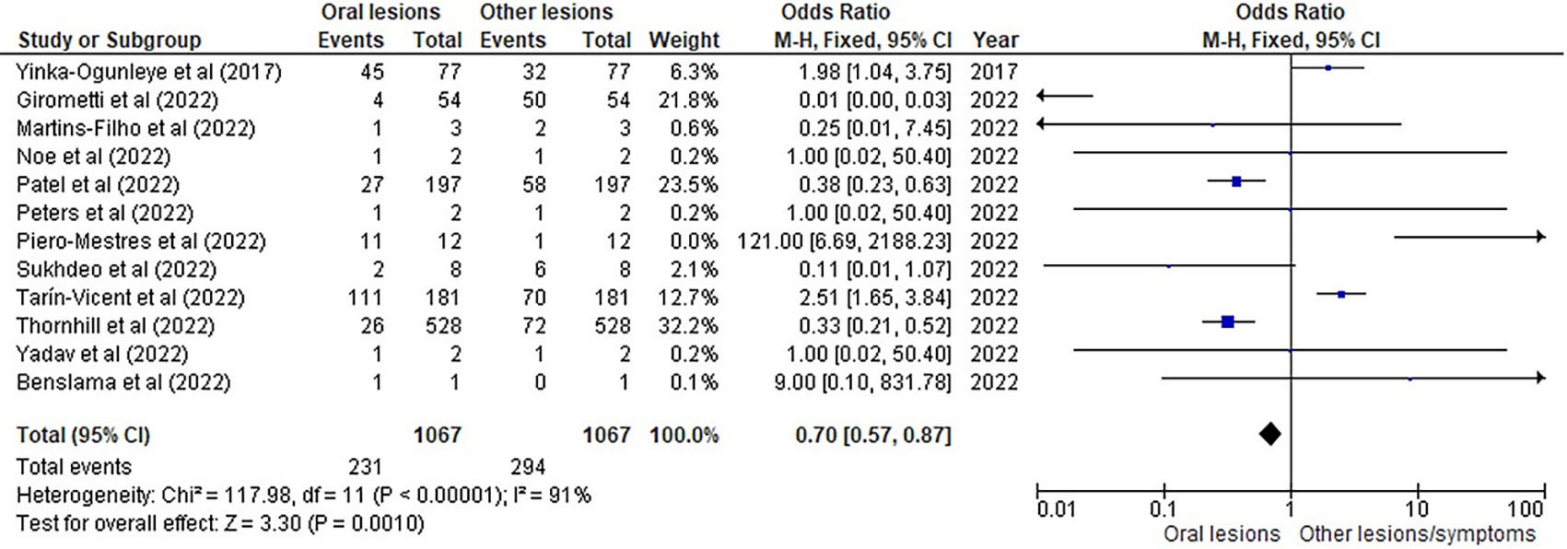
Incidence of oral lesions in monkeypox sufferers as compared to other lesions in the selected studies on the basis of the odds ratio represented on a forest plot.

**Figure 4.**
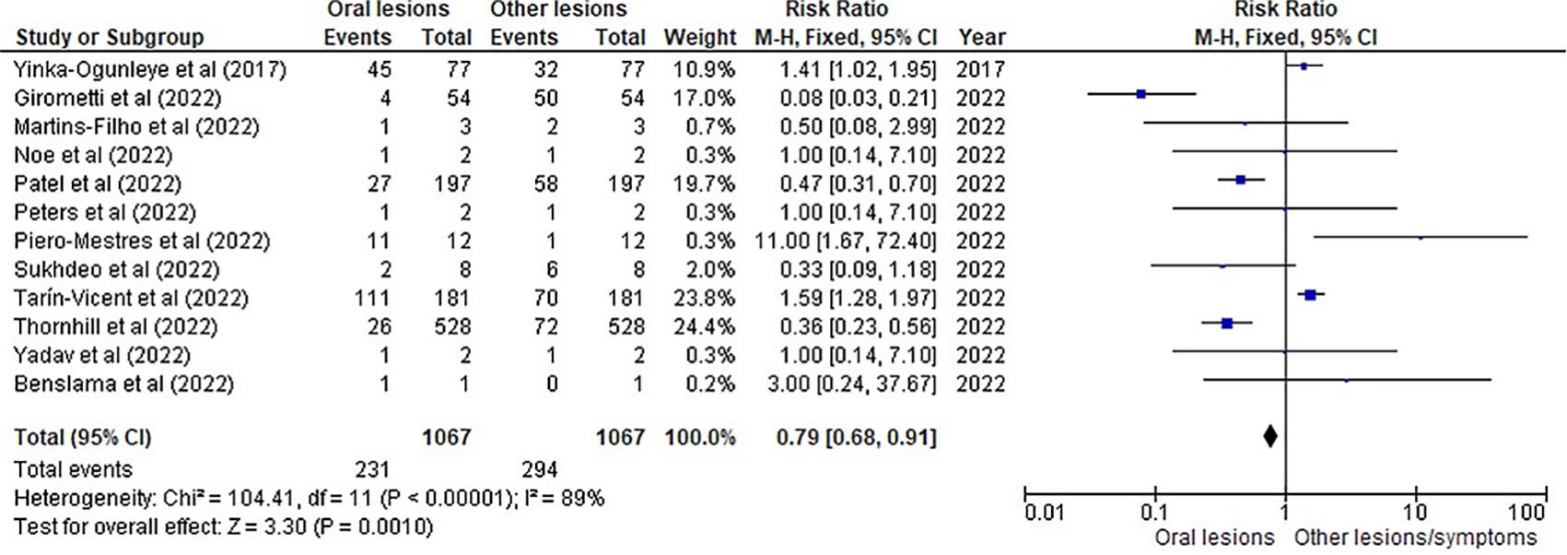
Incidence of oral lesions in monkeypox sufferers as compared to other lesions in the selected studies on the basis of the risk ratio represented on a forest plot.

**Figure 5.**
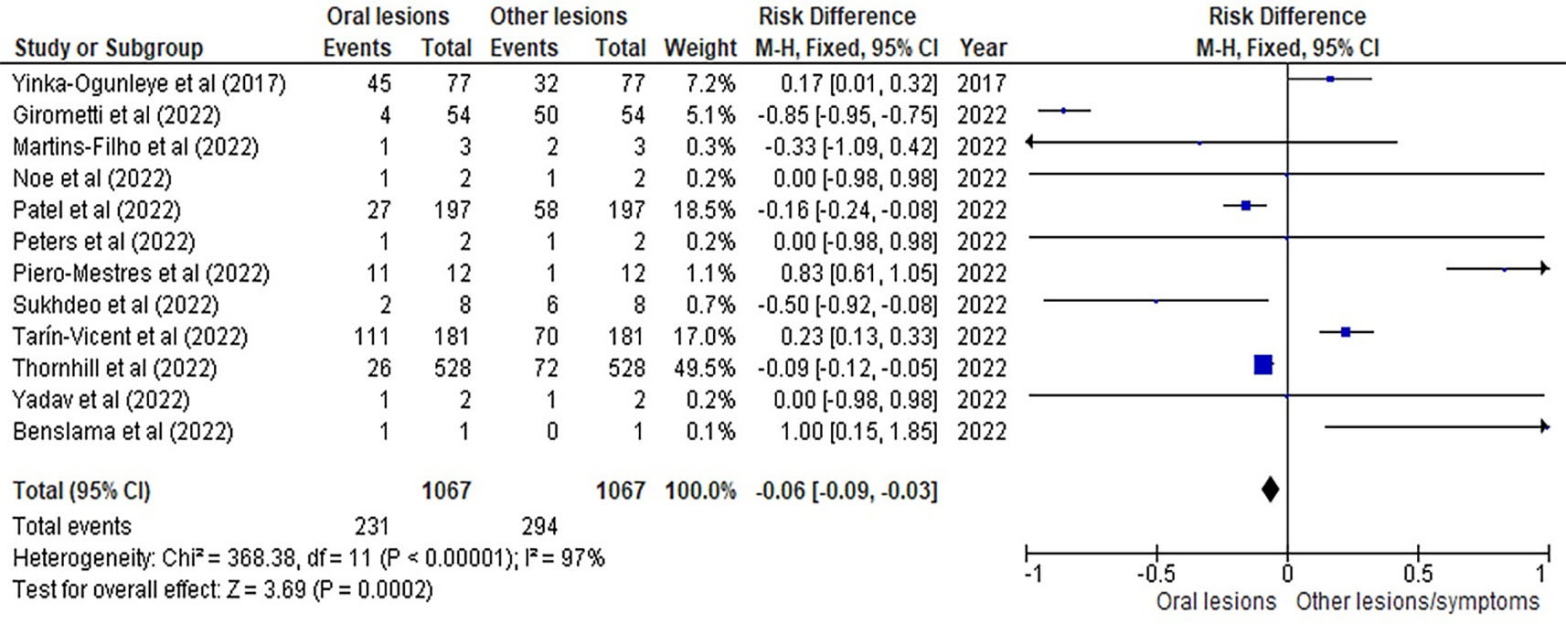
Incidence of oral lesions in monkeypox sufferers as compared to other lesions in the selected studies on the basis of the risk difference represented on a forest plot.

Table 3 as given below includes the findings of the systematic review as well as information on the 14 studies that were selected for the review. The results of the meta-analysis (conducted using RevMan 5 software) are displayed in Figures 3, 4 and 5 below in the form of a forest plot that reflects and ranks all the studies included in this systematic review. After data on the sample size, variables analysed, and various components of the investigations selected for our systematic review were entered into the Revman 5 programme, a forest plot illustrating the risk ratio, odds ratio, and risk difference was obtained as part of the meta-analysis for our study.

**Table 3.** Tabular representation of the studies used for this systematic review.

*Author and year of study*	*Sample size and mean age*	*Study design*	*Study description*	*Study inference*
Benslama et al (2022) ^12^	A 34 year old man	Case report	The patient had a 2-day history of fever and a mouth rash when they arrived. He also recorded mouth pain, swallowing issues, and headaches. He had a personal history of sexually transmitted diseases, particularly gonorrhoea and chlamydia genital infections.	Clinical examination revealed several mouth sores on the floor of the mouth and the top of the tongue, as well as bilateral laterocervical lymphadenopathy (figure). These oral lesions have a "cockade-like" pattern, with a white halo surrounding a central red ulcer. He didn't have any skin lesions when he arrived. Monkeypox was detected in swabs taken from lesions on the tongue using polymerase-chain reaction tests. Notably, the patient had not received a smallpox vaccination.
Girometti et al (2022) ^13^	54 (all males); mean age 41 years	Observational study	Of the 54 people, 30 (55%) had lymphadenopathy, while 4 (7%) had oropharyngeal lesions.	In this study, oral mucosal enanthema were more frequently recorded among unvaccinated individuals than among those who had received vaccinations. This condition can occur in more than 70% of cases.
Iamaroon et al (2022) ^14^	-	Literature review	At the time this study was being written, the monkeypox outbreak in non-endemic areas had spread to at least 47 nations and more than 4,100 new cases. In contrast to central and western Africa, the clinical characteristics in non-endemic locations are unusual. Monkeypox is mostly diagnosed based on clinical manifestations and laboratory tests.	Before the rash appears on the face and other areas of the body, it may first present as mouth lesions since the oral mucosa is frequently affected by this condition. It is possible for oral symptoms to appear before skin eruptions, indicating that dental professionals should be well-versed in the disease's characteristics.
Martins-Filho et al (2022) ^15^	3 (2 females); the females were 28 and 12 years respectively, with the male being 24 years old	Epidemiological case report	In a low-income area of Brazil, all 3 instances were identified between August 22 and August 26, 2022. Using real-time polymerase chain reaction, samples from skin lesions all tested positive for monkeypox DNA (RT-PCR).	Only the first of the three cases—involving a 28-year-old woman who saw the doctor 15 days after developing fever, asthenia, headache, and sore throat—was found to have early lesions in her oral mucosa. This case also had several pruritic, papulovesicular lesions on the limbs and trunk.
Noe et al (2022) ^16^	2 males	Observational study	In order to emphasise the significance of recent advances for medical experts around the world and to share further observations regarding human-to-human transmission in these cases, this paper details the first two cases of monkeypox (MPX) infections in Germany.	One patient described having trouble swallowing and having white patches on his tonsils. These oral sores on the tonsils were supposedly the patient's first outward sign of MPX. Such oral lesions have been previously identified for MPX in animal models as a component of the lymphatic tissue's involvement. These oral symptoms were absent in the second patient.
Patel et al (2022) ^17^	197 (all males); median age 38 years	Descriptive case series	Twenty seven participants presented with oral mucocutaneous manifestations without systemic symptoms.	Oral mucocutaneous lesions and systemic features were found to have a varied temporal relationship, which raises the possibility of a new clinical path for the illness.
Peters et al (2022) ^18^	2 (both males); 38 and 30 year old respectively	Case report	One of the patients noticed a sore, sensitive, "pimple-like" nodule on the tip of his tongue that grew larger before he went to the emergency room. A well-defined, tan-grey ulceration of the front tongue that was around 1.0 cm in size was found. However, the second person did not have any reported oral lesions.	The differential diagnosis for monkeypox oral symptoms frequently includes more prevalent inflammatory and viral diseases. If the lesion appears as a tan-grey ulceration involving the anterior tongue, traumatised ulcerations from biting may be taken into consideration.
Piero-Mestres et al (2022) ^19^	12 (all men); mean age 38.5 years	Clinical observation study	Real-time PCR was used to gather and evaluate 147 clinical samples from 12 individuals at various times. All instances had monkeypox DNA in their saliva, sometimes with high virus levels.	11 out of 12 patients reported experiencing a generalised systemic syndrome, including fever, myalgia, general malaise, and mouth ulcers. Oral lesions were found in at least one area of the oral cavity in half of the individuals.
Samaranayake et al (2022) ^20^	-	Case review	The information was gleaned from the most recent literature, primarily from the databases of the World Health Organization and the Centres for Disease Control and Prevention, and covered the aetiology, modes of transmission, signs and symptoms, diagnosis, and management, as well as the risk of occupational transmission in dental settings.	Clinicians should be aware that the disease typically manifests as macules and ulcers on the oral mucosa before the characteristic skin lesions. Patients should be isolated and referred when necessary, especially when a local outbreak is present. Standard, contact, and droplet infection control measures should also be implemented.
Sukhdeo et al (2022) ^21^	8 (all males); age range from 25-56 years	Observational study	In this study, clinical photos are provided to demonstrate the range of cutaneous and mucocutaneous lesions that eight patients with HMPX (whose diagnosis was supported by real-time polymerase chain reaction) presented with while receiving therapy in Toronto, Canada, between May and July 2022.	A primary tongue lesion was present in one patient. The 5 mm ulcer on the right tip of the tongue was painful, covered in purulence, and encircled by oedema. There was a localised swelling surrounding another ulcer on the left posterior part of the dorsal tongue, which is not visible in this image. The patient's initial clinical presentation consisted of these ulcers, which were a primary crop of lesions. On another patient's right upper lip, there was an ulcer. The 12 mm peripheral erythematous ulcer was not painful or pruritic.
Tarín-Vicent et al (2022) ^22^	181 (all males); median age 37 years	Observational cohort study	Demographics, smallpox vaccination, HIV status, exposure to monkeypox, travel, mass gathering attendance, risk factors for STIs, sexual behaviour, signs and symptoms at initial presentation, virological results at multiple body sites, co-infection with other STIs, and clinical outcomes 14 days later were all outcomes assessed in all participants with a confirmed diagnosis.	Lesions in the oral and perioral area were present in 78 people (or 43%). 70 (39%) of the patients experienced difficulties that required medical attention, including 19 (10%) tonsillitis, 6 (3%) abscesses, and 8 (4%) exanthems.
Thornhill et al (2022) ^23^	528 (all males)	International descriptive case series	26 people reported that their first symptoms were oropharyngeal, including pharyngitis, odynophagia, epiglottitis, and oral or tonsillar lesions.	A variety of dermatologic and systemic clinical symptoms were present in monkeypox manifestations. The oral route (in the form of droplets), close or direct contact with skin lesions are the routes by which the monkeypox virus is spread.
Yadav et al (2022) ^24^	2 (both males); 35 years and 31 years old respectively	Case report	One of the patients experienced several vesicular rashes in the lips and oral cavity, which led to an oedematous upper lip. None of the significant mouth lesions were visible to the other patient.	Oral lesions are an important diagnostic marker in cases of monkeypox.
Yinka-Ogunleye et al (2017) ^25^	122 (84 males); median age 29 years	Epidemiological and clinical study	A form of oral vesicular rash appeared in all 122 confirmed or probable cases, and 45 (58%) of the 77 confirmed patients also experienced sore throats.	A rash on the tongue and mucous membranes, as well as any sores or lesions on the tongue, in the oral cavity, or on the corners of the mouth, are common early signs of monkeypox.

Figures 3, 4 and 5 represent the forest plots obtained after meta-analysis of the incidence of the oral lesions in patients of monkeypox that were a part of the studies that were selected for our systematic review.

## Discussion

Many diseases, including chickenpox, measles, bacterial skin infections, scabies, syphilis, and medication-induced allergies, share characteristics of the mucocutaneous lesions with monkeypox. ^26^ Based solely on the clinical appearance, it might be challenging to discern between these disorders in some cases. When comparing monkeypox to chickenpox or smallpox, lymphadenopathy during the prodromal stage might be used to make the distinction. Healthcare professionals should gather a sample if monkeypox is suspected so that it can be further examined using polymerase chain reaction (PCR), a method that has strong specificity and sensitivity for finding the monkeypox virus. ^27^ The fluid from vesicles, pustules, or dry crusts should be collected, preserved in a dry, sterile tube without viral transport media, and kept cold. An optional method for making the diagnosis is a biopsy. ^28^

As evident by the finding mentioned in Table 3 depicting the selected studies, oral lesions are primarily present at the start of the monkeypox affliction. For example, in the study by Tarín-Vicent et al, ^22^ lesions in the oral and perioral area were present in 78 people (or 43%). In all the studies, oral lesions were present in some form or the other (as ulcers or vesicular rash most commonly around the tongue or lower lip area) during the prodromal stage of monkeypox which was accompanies by symptoms such as fever, myalgia, and general malaise and, in some cases, a pruritic rash. The case reviews by Iamaroon et al ^14^ and Samaranayake et al ^20^ also provide with literature evidence of oral lesions in monkeypox patients. One of the documented cases ^29^ in these reviews mention of a male patient who was a returning tourist from Nigeria experienced right cervical lymphadenopathy, multiple 2-4-mm pustules, and central umbilication of the skin, particularly on the face, neck, and hands. The oral lesions were described as a single intact pustule on the lower labial mucosa and a few round 2-3 mm erosions on the mucosa, indicating that the original vesicles or pustules have already broken off. In the study by Girometti et al, ^13^ oral mucosal enanthema were more frequently recorded among unvaccinated individuals than among those who had received vaccinations. This condition can occur in more than 70% of cases. This might represent the aspect of oral lesions occurring more in unvaccinated individuals which might prompt strengthening of the already stringent vaccination laws around the world especially in terms of traveling. Speaking of traveling, seven of the 14 studies that we selected for our review involved individuals who were traveling from one country to another which again warrants a separate investigation into the transmission of monkeypox or monkeypox-like infectious diseases within a particular defined area (such as a city, state or country) and how it might differ from the outcomes obtained in our investigation.

Figures 3, 4 and 5 represent the meta-analysis results of our investigation. Although the heterogeneity levels in the three different assessments are particularly high (91%, 89% and 97%), it is due to the fact that the studies that were selected had sample sizes that were lesser than what could be considered ideal and more importantly, the methodological differences in the studies selected contribute to the high levels of heterogeneity of the forest plots. The fixed effect model was employed in the odds ratio, risk ratio and the risk difference assessments so as to keep uniformity in terms of our interpretations and reduce bias that occurs resultantly after selection of studies with such variations in methodology and sample sizes.

The nasopharyngeal swab bio sample has been the gold standard for the detection of severe acute respiratory syndrome coronavirus 2 since the start of the COVID-19 pandemic (SARS-CoV-2). Saliva has recently become a practical and affordable bio fluid for COVID-19 diagnostics and may someday take the place of a nasopharyngeal swab. ^30^ Saliva collection requires no specialised equipment, is non-invasive, and uses a straightforward approach. Since monkeypox infection frequently appears in the oral cavity, patient saliva may contain monkeypox virions and hence might be used as a bio sample to identify the virus. These patients will greatly benefit from further research to validate the use of saliva for monkeypox diagnoses. ^31^ Vesicular or pustular lesions are the terms used to characterise monkeypox oral symptoms. The ulceration occurs following the vesicle or pustule rupture. Monkeypox patients' lesions may mirror those caused by other viral diseases of the oral cavity, such as COVID-19, chickenpox, measles, measles-mumps-rubella, measles, and herpes zoster. Vesiculobullous lesions predominate in monkeypox oral lesions. The vesicles/bullae readily separate, resulting in numerous uncomfortable, superficial sores on the oral mucosa and lips. Recurrent lesions are more confined and frequently affect the keratinized oral mucosa and lip vermilion. ^32^

Fortunately, the majority of people with monkey pox infection recover on their own. To reduce gastrointestinal fluid losses, those with gastrointestinal symptoms (such as vomiting or diarrhoea) will need oral or intravenous rehydration. Several antivirals have been licenced for the management of smallpox based on animal models, but they may also be beneficial in treating monkeypox infections. Human dose studies for these medications have been carried out, but their effectiveness has not been fully explored. The first antiviral approved for the treatment of smallpox in adults and children weighing at least 3 kg is tecovirimat, and it is regarded as the preferred method of care. ^33^ Dual therapy with tecovirimat and brincidofovir may be utilised in patients with advanced illness. By blocking the last steps in viral maturation and release from the infected cell, the viral envelope protein VP37—by which Tecovirimat functions—inhibits the transmission of the virus within an infected host. ^34^ Although its effectiveness in treating monkeypox in people has not been investigated, investigations on animals treated with tecovirimat at various illness phases have shown better survival from lethal monkeypox virus infections in comparison to animals given with a placebo. ^35^^–^^36^ The side-effect profile of the placebo was generally identical to that of tecovirimat in an enlarged safety study with 359 human volunteers given tecovirimat. Since June 2021, brincidofovir has also been authorised for the treatment of monkeypox in the US. ^37^ An oral counterpart of the injectable medicine cidofovir, brincidofovir, may have a better safety profile than cidofovir, such as reduced renal damage. ^38^ These medications function by preventing viral DNA polymerase. ^39^ The effectiveness of brincidofovir against orthopoxvirus infections has been established, despite the paucity of studies examining its usage in treating monkeypox infections in animal models. ^40^ The FDA has authorised the hyperimmune globulin known as Vaccinia Immune Globulin (VIG) for the treatment of specific vaccine-related side effects. ^41^ These include vaccinia infections in people with skin disorders, aberrant infections brought on by the vaccinia virus, progressive vaccinia, severe widespread vaccinia, and eczema vaccinatum (except in cases of eye-related infections). ^41^

As far as limitations go, our systematic review had a few to being with. For starters, the sample size observed in our selected studies were fewer than what would be considered ideal, but, since the number of articles which had documented the oral manifestations observed in patients suffering from monkeypox are very scarcely available, we selected the ones best suited for our objectives. Additionally, prior to the outbreak in May 2022, monkeypox had a limited clinical relevance, therefore it was frequently overlooked in the differential diagnosis. Other infectious illnesses could be in the differential diagnosis list. The countries of Central and Western African regions, where this disease is endemic, do not have a lot of data available on monkeypox due to the extremely limited number of studies performed, which as such necessitates the importance of documenting monkeypox and its effects observed in the form of symptoms such as the occurrence of oral lesions.

## Conclusion

All the 14 articles selected in this review have reported the incidence of oral lesions in monkey pox sufferers, in at least the beginning stages of the disease, which have then blown up into full-fledged symptoms. As such, the identification and treatment of this illness may be aided by the seldom observed oral features of monkeypox infection. Acute onset oral signs (as seen in nearly all our selected studies) should be differentially diagnosed for monkeypox, especially in people who are more likely to experience this ailment. It is crucial to remember that recognising and identifying oral lesions in patients with monkeypox paves the way for additional research, effective care, and the prevention of cross-infection between patients and medical staff.

## Author contributions

Conceptualization, HA, AD, FB, NM, FA, OM, KPS, MAS, HA, MKA; methodology, HA, AD, MKA; software, HA, AD, MKA; validation, HA, AD, MKA; formal analysis, HA, AD, MKA; investigation, HA, AD, MKA; resources, HA, AD, MKA; data curation, HA, AD, MKA; writing—original draft preparation HA, AD, FB, NM, FA, OM, KPS, MAS, HA, MKA; writing— review and editing. HA, AD, FB, NM, FA, OM, KPS, MAS, HA, MKA; visualization, HA, AD, MKA; project administration, HA, AD, MKA; funding acquisition, none. All authors have read and agreed to the published version of the manuscript.

## Reporting guidelines

Figshare. PRISMA flowchart. DOI: https://doi.org/10.6084/m9.figshare.23626704.v1

Figshare. PRISMA checklist. DOI: https://doi.org/10.6084/m9.figshare.23626695.v1

## Data Availability

All data are available within the manuscript.
