# Supplementary material for: Monkeypox and oral lesions associated with its occurrence: a systematic review and meta-analysis
Source: F1000Res. 2024 Mar 1;12:964. Originally published 2023 Aug 10. [Version 2] doi: 10.12688/f1000research.137363.2 (PMC11153993; doi:10.12688/f1000research.137363.2)
Supplement: Peer review history

# Reviewer report: Reviewer response for version 2

**DOI:** 10.5256/f1000research.162619.r278735

**Published:** 2024-06-05

**Authors:** Mohamed LOUNIS

**Affiliations:** 1 University of Ziane Achour, Djelfa, Algeria

I would like to thank the authors for this interesting manuscript. However, after reviewing, I found that the manuscript has shown some gaps that have affected its quality:

 1. The manuscript requires an extensive language editing. The grammar and the sequencing of the ideas is a lacking character of this manuscript.

 The introduction of the abstract did not provide information in relation with the subject and has not allowed to define the hypotheis.

 The results are not well described in the abstract.

 Introduction:

 in the first sentence : the mpox virus is a zoonotic agent (not a disease)

 The term mpox should be defined in the beginning of the sentence, not later.

 The introduction should be revised. it seems to be composed of multiple separated sentences (try to correlate between the sentences).

 Also, it has not alowed to define the hypotheis of the work.

 The results are very simplistically presented, despite the interesting results provided. They were not practically described either in the results or in the discussion. The same is true for the discussion, which contains two (simple paragraphs (2 and 3) and a second part (paragraphs 3 and 4), which has practically no relation to the work.

 The statisticall analysis are not fully described.

 At last, I have a concern that really bothered me, which is the fact that you included case reports and reviews in this systematic review

Are the rationale for, and objectives of, the Systematic Review clearly stated?

Yes

Is the statistical analysis and its interpretation appropriate?

Partly

If this is a Living Systematic Review, is the ‘living’ method appropriate and is the search schedule clearly defined and justified? (‘Living Systematic Review’ or a variation of this term should be included in the title.)

Not applicable

Are sufficient details of the methods and analysis provided to allow replication by others?

No

Are the conclusions drawn adequately supported by the results presented in the review?

Yes

Reviewer Expertise:

infectious diseases, public health

I confirm that I have read this submission and believe that I have an appropriate level of expertise to confirm that it is of an acceptable scientific standard, however I have significant reservations, as outlined above.

# Reviewer report: Reviewer response for version 2

**DOI:** 10.5256/f1000research.162619.r252060

**Published:** 2024-03-12

**Authors:** Bruno Hernaez

**Affiliations:** 1 Centro de Biologia Molecular Severo Ochoa (CBMSO) - Consejo Superior de Investigaciones Cientificas (CSIC), Nicolás Cabrera, Madrid, Spain

I thank the authors for updating the nomenclature and naming the disease as "mpox", however I consider that this should also apply to the title to meet the WHO recomendations. Thus the title should be "Mpox and oral lesions associated with its occurrence: a systematic review and meta-analysis"

Are the rationale for, and objectives of, the Systematic Review clearly stated?

Yes

Is the statistical analysis and its interpretation appropriate?

I cannot comment. A qualified statistician is required.

Are sufficient details of the methods and analysis provided to allow replication by others?

Yes

Are the conclusions drawn adequately supported by the results presented in the review?

Yes

Reviewer Expertise:

poxvirus immune evasion

I confirm that I have read this submission and believe that I have an appropriate level of expertise to confirm that it is of an acceptable scientific standard, however I have significant reservations, as outlined above.

# Reviewer report: Reviewer response for version 1

**DOI:** 10.5256/f1000research.150522.r208362

**Published:** 2023-09-26

**Authors:** Bruno Hernaez

**Affiliations:** 1 Centro de Biologia Molecular Severo Ochoa (CBMSO) - Consejo Superior de Investigaciones Cientificas (CSIC), Nicolás Cabrera, Madrid, Spain

The present systematic review addresses ﻿the association between mpox (formerly monkeypox) and the presence of oral lesions in the patients. This is clearly an interesting topic given the relevance of this disease, especially since the 2022 outbreak affecting non-endemic regions. However, I have some comments and concerns that hopefully will contribute to improve this manuscript.

1. In my opinion, the introduction can be improved. For instance, Monkeypox virus is NOT an uncommon zoonotic disease as the authors state in their first sentence of the introduction.  Monkeypox virus is the name for the virus causing the disease monkeypox. In addition, after the 2022 outbreak there is consensus to follow the WHO nomenclature recommendations: the disease is now “mpox” and the virus is “mpox virus (MPXV)”. Please change through the entire text.
2. The authors state in Table 1: “﻿ *There have been no reports of patient deaths from non-endemic regions up to this point*”. This needs an update since at this moment 157 deaths have been reported (file:///Users/bh/Downloads/20230919_mpox__external-sitrep-28.pdf).
3. Viable virus in saliva was first described by our group in a study published in The Lancet Microbe (doi: 10.1016/S2666-5247(22)00291-9). The incorporation of this reference would support the affirmation “ *﻿These findings imply that the virus can be carried in the saliva and spread via oral-skin and/or oral-anogenital contact*”.
4. In line with this, I wonder why this study is not included in the present review. The study by Hernaez et al (Monitoring monkeypox virus in saliva and air samples in Spain: a cross-sectional study) included 44 confirmed patients with mpox from the 2022 outbreak in Madrid (Spain), and the clinical symptoms (including the presence of oral lesions) can be found in an exhaustive table as Supplementary material. In my opinion the authors should contemplate its incorporation now, perhaps by adding the term “saliva” to the search strategy.

Are the rationale for, and objectives of, the Systematic Review clearly stated?

Yes

Is the statistical analysis and its interpretation appropriate?

I cannot comment. A qualified statistician is required.

Are sufficient details of the methods and analysis provided to allow replication by others?

Yes

Are the conclusions drawn adequately supported by the results presented in the review?

Yes

Reviewer Expertise:

poxvirus immune evasion

I confirm that I have read this submission and believe that I have an appropriate level of expertise to confirm that it is of an acceptable scientific standard, however I have significant reservations, as outlined above.

## References

[1] : Monitoring monkeypox virus in saliva and air samples in Spain: a cross-sectional study. *Lancet Microbe* .2023;4(1) : 10.1016/S2666-5247(22)00291-9 e21-e28 10.1016/S2666-5247(22)00291-9 36436538 PMC9691259

# Reviewer report: Reviewer response for version 1

**DOI:** 10.5256/f1000research.150522.r195852

**Published:** 2023-08-30

**Authors:** Gururaj Arekeri

**Affiliations:** 1 Department of Head and Neck Surgical Oncology and Robotic Surgery, HealthCare Global (HCG) Cancer Centre, Bangalore, Karnataka, India

Thank you for asking me to review the article "Monkeypox and oral lesions associated with its occurrence: a systematic review and meta-analysis":

 Comments:

- The article addresses an important and timely topic given the recent global outbreak of monkeypox cases. Understanding the oral manifestations can aid healthcare providers in early detection and diagnosis.
- The authors clearly state the objectives to establish the association between monkeypox and oral lesions through a systematic review and meta-analysis. 
- This review synthesizes important evidence on monkeypox oral manifestations from multiple case reports and observational studies. It will help inform clinicians in this critical area.
- The methods are sound and align with guidelines for rigorous systematic reviews. The number of studies included is understandable given monkeypox's emerging nature.
- The article is comprehensive in summarizing prior literature and relevant clinical details on oral lesions in monkeypox. This will aid differential diagnosis.
- The authors note study limitations appropriately and suggest implications for future research. 
- The topic is highly relevant currently. This review makes a valuable contribution to the literature on this unfolding outbreak.

Overall, I recommend approval of this well-conducted systematic review for indexing.

Are the rationale for, and objectives of, the Systematic Review clearly stated?

Yes

Is the statistical analysis and its interpretation appropriate?

Yes

Are sufficient details of the methods and analysis provided to allow replication by others?

Yes

Are the conclusions drawn adequately supported by the results presented in the review?

Yes

Reviewer Expertise:

OMFS

I confirm that I have read this submission and believe that I have an appropriate level of expertise to confirm that it is of an acceptable scientific standard.
